# Melanocortin Peptides: Potential Targets in Systemic Lupus Erythematosus

**DOI:** 10.1007/s10753-014-0029-5

**Published:** 2014-10-17

**Authors:** Lisa Carole Loram, Melissa Elizabeth Culp, Erin Corey Connolly-Strong, Sheila Sturgill-Koszycki

**Affiliations:** Mallinckrodt Pharmaceuticals (formerly Questcor), 26118 Research Road, Hayward, CA 94545 USA

**Keywords:** melanocortin, systemic lupus erythematosus, adrenocorticotropin, immune

## Abstract

Systemic lupus erythematosus (SLE) is a systemic autoimmune disease resulting in loss of self-tolerance with multiple organs, such as the kidney, skin, joints, and the central nervous system (CNS), being targeted. Numerous immunosuppressant therapies are currently being used for the treatment of SLE, but their clinical utility is somewhat variable because of the clinical heterogeneity. Melanocortins are a family of peptides derived from the common precursor protein pro-opiomelanocortin. These multifunctional peptides activate five subtypes of melanocortin receptors expressed on immune, skin, muscle, bone, and kidney cells and cells within the CNS. Melanocortin peptides have demonstrated a variety of biologic actions including immunomodulation, melanogenesis, and renoprotection. This review aims to introduce the melanocortin system and explore the mechanisms by which they may be beneficial in diseases such as SLE.

## INTRODUCTION

Systemic Lupus Erythematosus (SLE) is characterized by the loss of self-tolerance and chronic inflammation that manifest in organs such as the skin, kidney, and central nervous system (CNS). High levels of morbidity and mortality are associated with SLE, resulting from damage in a variety of organs. A hallmark of SLE is the presence of autoantibodies, which can be directed against nuclear and cytoplasmic antigens. These observations have led to a strong interest in therapeutically targeting the immune system. A number of immunosuppressant therapies are currently used for the treatment of SLE, including synthetic glucocorticoids, anti-malarials, and other immune suppressants, including the B cell-targeted therapy Benlysta® (belimumab) [1]. Belimumab is a human monoclonal antibody that inhibits B cell activating factor (BAFF, a B cell survival factor), which subsequently reduces immature B cell populations and serum autoantibody levels [2]. Since the immune complex deposition within end organs is the key contributor to end organ damage, targeting B cell populations is an ideal strategy. Other potential new therapies are designed to target immune cell function known to be important in the pathogenesis of SLE, including targeting B cell survival, various lymphocyte receptors, and inhibiting interferon alpha (IFN-α).

The immune hyperactivity of SLE can be inferred systemically by elevations in levels of cytokines and upregulation of their associated receptors [3]. For example, interleukin (IL)-17 is a cytokine that induces the production of inflammatory and tissue-damaging molecules [4]. Serum or plasma levels of IL-17, as well as expansion of IL-17-producing T cells in the peripheral blood, are both elevated in patients with SLE [4]. The commitment of naïve T lymphocytes (CD4^+^) to the Th17 lineage is attributed to, among other mechanisms, the presence of both transforming growth factor (TGF)-β and IL-6 [4]. The concentration of TGF-β1 also was found to be decreased in SLE patients compared to healthy controls [5].

Three of the 11 ACR criteria for SLE relate to photodistribution and skin manifestations [6], and skin manifestations are the most frequently observed symptom in SLE patients. The underlying pathogenesis of cutaneous SLE is thought to involve antibody deposition at the dermal-epidermal junction, keratinocyte hyperproliferation with early differentiation, and premature terminal differentiation and inflammatory cell infiltrates [6]. Photosensitivity is one of the major manifestations of SLE, and cutaneous lupus lesions can be triggered by sunlight exposure possibly from induction of UV-induced apoptosis. The mean number of p53-positive (apoptosis marker) keratinocytes in the skin of SLE patients with cutaneous manifestations is significantly higher than that of SLE patients with no cutaneous manifestations or healthy volunteers [7].

Beyond circulating immune cell changes and inflammatory mediators upregulated in the periphery, immune complex deposition occurs in several organ systems, resulting in inflammation and organ damage. The key organs include the kidneys, brain, and skin. Lupus nephritis is one of the most severe manifestations of SLE and is associated with considerable morbidity and mortality. Up to 35 % of patients with SLE present with signs of lupus nephritis at the time of diagnosis, and up to 60 % of patients develop lupus nephritis within the first 10 years of their disease. The presence of lupus nephritis significantly decreases the chance of 10-year survival [8]. The development of nephritic lesions in SLE patients is likely from a combination of increased inflammatory activity, B cell and T cell dysfunction, aggregate antibody complex formation, and subsequent complement activation [9].

CNS lupus is area of high unmet need, as many current treatment options and studies exclude CNS lupus. Clinical manifestations of CNS lupus are broad, ranging from psychosis and stroke to subclinical cognitive dysfunction [10]. The pathogenesis of CNS lupus is poorly understood, although anti-phospholipid antibodies, vasculopathy and vasculitis, and local inflammatory processes have been implicated. Additionally, intrathecal levels of glial fibrillary acidic protein (GFAP) were increased in SLE patients with CNS involvement compared to SLE without overt CNS disease. GFAP is an intermediate filament protein expressed by astrocytes, and cerebrospinal fluid levels of GFAP in SLE patients are significantly correlated with MRI abnormalities [11]. Therefore, damage to glial cells within the brain may contribute to the pathogenesis of CNS lupus.

The immune processes and organs affected by SLE may be viable therapeutic targets of steroid-dependent and steroid-independent effects of adrenocorticotropin hormone (ACTH). ACTH was approved in the early 1950s and was used interchangeably with prednisone for numerous inflammatory diseases, including rheumatoid arthritis and SLE. ACTH is well known to stimulate endogenous cortisol production and is a key component of the hypothalamic-pituitary adrenal axis. However, ACTH also has activity at melanocortin receptors outside of the adrenal gland where biologic activity extends beyond steroidogenesis. Melanocortins are endogenously produced and have demonstrated anti-inflammatory and organ-protective effects [12–14]. This review aims to provide a broader appreciation of the melanocortin peptides, highlight their anti-inflammatory effects on varied immune cell subsets, and stimulate discussion on the possible role(s) for melanocortins in the treatment of SLE.

## MELANOCORTIN PEPTIDES AND RECEPTORS

**Fig. 1:**
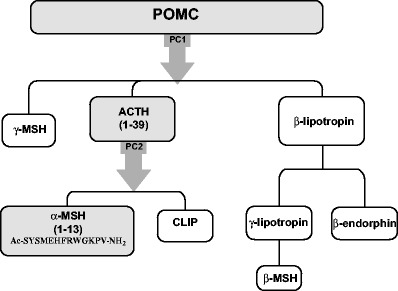
Pro-opiomelanocortin peptide and the post-translational cleavage products [15].

The melanocortin peptides include ACTH, alpha-melanocyte-stimulating hormone (α-MSH), β-MSH, and γ-MSH, all of which are cleaved from a common precursor polypeptide, pro-opiomelanocortin (POMC, see Fig. 1 [15]) [16]. POMC is predominantly synthesized and processed in the pituitary gland, although small amounts of this precursor protein are also synthesized and processed in extrapituitary sites, including immune and skin cells [17]. POMC is proteolytically cleaved into numerous fragments, including ACTH. ACTH is a 39-amino acid peptide that can be further cleaved to generate α-MSH, which is the first 13 amino acids of ACTH [17]. All melanocortin peptides, ACTH, α-MSH, β-MSH, and γ-MSH, have a core sequence of four amino acids (His-Phe-Arg-Trp) that are required for receptor binding [18]. Synthetic melanocortin peptides have been developed, including ACTH (1–24) also named tetracosactide. Of the synthetic agents, tetracosactide is used clinically outside of the USA for diseases other than lupus, while the other synthetic melanocortin analogues are being tested in preclinical models or in early phase development.

Melanocortin peptides bind with different affinities and selectivity to five identified melanocortin receptor subtypes (melanocortin receptors (MC1–5R) that are distributed on multiple cell populations distributed throughout the body [19]. The MC1R has a well-characterized involvement in skin and hair pigmentation and immune cell regulation [20], while ACTH is the only melanocortin peptide that activates MC2Rs in the adrenal gland to stimulate glucocorticoid synthesis and release [21]. MC3R regulates immune cell function, and both MC3R and MC4R have roles in energy homeostasis and regulate neuronal interactions with autonomic functions [22–24]. The MC5R is expressed on a broad range of tissues throughout the body; however, its function remains to be fully elucidated, although this receptor has been implicated in sebaceous gland function and in the stress response, based on its primary expression in exocrine glands [25].

Melanocortin receptors are transmembrane, G protein-coupled receptors (GPCRs) that regulate diverse intracellular signal transduction mechanisms, including cyclic adenosine monophosphate (cAMP) and protein kinase A (PKA) signaling pathways [26, 27]. One of the downstream effects of the melanocortin intracellular cascade is inhibition of the canonical pro-inflammatory transcription factor nuclear factor kappa B (NF-κB). This effect of melanocortin receptor activation appears to be dose and time dependent and involves stabilization of the cytosolic inhibitory IκB protein, which retains NF-κB within the cytoplasm, thereby preventing NF-κB from translocating to the nucleus and driving pro-inflammatory mediator transcription [28, 29]. Other intracellular cascades can be activated by melanocortin receptor signaling, for example, ERK1/2 signaling, Jak/STAT signaling, and AP-1 transcription [24, 30, 31]. Cyclic AMP response element-binding protein (CREB) can also be activated by melanocortin receptor activation [26] resulting in improved cell survival from metabolic and oxidative stress [13]. For a more comprehensive review on signaling, see previous reviews [12, 13, 15].

## MC2R AND STEROIDOGENESIS

In the classic hypothalamic-pituitary adrenal axis, ACTH is released from the anterior pituitary gland and is the primary systemically released endogenous melanocortin peptide that binds to and activates MC2R, which is predominantly located in the adrenal cortex, especially in the zona glomerulosa and zona fasciculata [21]. MC2R adrenocortical receptor signaling and subsequent release of cortisol is a well-characterized process, which involves MC2R signaling through cAMP and PKA to stimulate movement of cholesterol into the mitochondria and increases the expression of steroidogenic enzymes resulting in steroidogenesis [21]. Interestingly, MC2R appears to upregulate its own expression upon stimulation by ACTH [32] but does require an accessory protein to mobilize the receptor to the cell surface [21].

The potent anti-inflammatory actions of glucocorticoids in the treatment of SLE have been studied since the 1950s [1]. In that era, it was assumed that the only clinical benefit of ACTH was to stimulate glucocorticoid release [33, 34]. However, subsequent research has revealed that ACTH also binds to and activates four other melanocortin receptor subtypes, which have biologic effects beyond steroidogenesis. The remainder of the review will focus on the potential therapeutic benefit of targeting these other four melanocortin receptors, MC1R, MC3R, MC4R, and MC5R, which provide non-steroidogenic effects of melanocortin peptides.

## POTENTIAL EFFECT OF MELANOCORTINS ON IMMUNE CELLS

Melanocortin receptor signaling leads to anti-inflammatory and immunomodulatory effects in a variety of *in vitro* and *in vivo* models (Table 1). Below, we explore the impact of melanocortin signaling on immune cell function.

**Table 1:** Immunomodulating Effects of Melanocortins

Regulation	Class	Specific molecules	References
Downregulation	Proinflammatory	IL-1, IL-6, TNF-α	[35, 36] [37, 38] [35, 37–39]
Downregulation	Immunomodulatory	IL-2, IL-4, IL-13, IFN-γ, IL-17	[40] [16, 41, 42] [16, 43] [42] [44]
Downregulation	Co-stimulatory receptors	CD40, CD86 (CD27)	[45] [46]
Downregulation	Antigen presentation	MHC class I, CD1a	[46] [47]
Downregulation	Adhesion molecules	ICAM-1, VCAM-1, E-selectin	[48, 49]
Downregulation	Effectors	Nitric oxide, Prostaglandin E, Reactive oxygen intermediates, Myeloperoxidase, MMP-1, MMP-3, and MMP-13	[50, 51] [52, 53] [38, 54] [43] [37, 55]
Downregulation	Chemokines/growth factors	IL-8/IL-8R CCL2, CXCL1	[37, 38, 43] [56] [56]
Downregulation	Innate signaling mechanisms	TLR4 CD14	[57] [58]
Upregulation	Immunomodulatory	IL-10, TGF-β, LAP	[29, 59] [60] [60]
Upregulation	Protective mechanisms	Superoxide dismutase 2, heme oxygenase 1, Bcl-2 Bcl-xL	[61] [61] [53, 62] [63]

### Macrophages

Melanocortin peptides have been shown to reduce macrophage release of pro-inflammatory mediators and increase anti-inflammatory cytokines. An *in vitro* study of a mouse macrophage cell line demonstrated that treatment with ACTH increased the anti-inflammatory cytokine IL-10 via PKA pathway activation [29]. In addition, α-MSH significantly reduced the *in vitro* release of pro-inflammatory cytokines (IL-1β, IL-8, and TNF-α) and macrophage nitric oxide production [29]. Furthermore, AP214, an α-MSH analogue with high affinity for MC1R, MC3R, and MC5R, increases macrophage phagocytosis, as detected by increases in uptake of zymosan (surface ligand of fungi) particles and human apoptotic neutrophils by macrophages. These melanocortin peptide effects on macrophage function might be mediated through MC3R, as MC3R null macrophages do not demonstrate increase in phagocytosis after AP214 treatment [76]. Regardless of the primary melanocortin receptor or signaling pathway affected, a decrease in inflammatory cytokine release from macrophages could be relevant in SLE.

Nitric oxide (NO) is a very versatile component of the immune system, with involvement in both cytotoxic and regulatory functions. The production of reactive nitrogen and oxygen intermediates is an important piece of the innate immune response. The overproduction of NO has been reported in the setting of active SLE [77]. Two studies have demonstrated the downregulation of inducible nitric oxide synthase (iNOS) in immune cells treated with melanocortin peptides. α-MSH inhibits NO release in cultured macrophage cell lines via NF-κB-dependent and NF-κB-independent pathways [29]. The inhibitory effects of α-MSH on NO levels in cultures of stimulated murine microglia, resident phagocytic cells of the CNS, were attributed to reduced iNOS expression [52]. These results demonstrate the potent effects of melanocortin peptides on the inflammatory production of NO.

### B Lymphocytes

B cells are an important therapeutic target in SLE as the producer of autoantibodies. The effect of ACTH on B cells was evaluated using the NFB/W F1 mouse model of SLE-like disease manifestations, including proteinuria. Mice were treated with either ACTH (as found in a highly purified gel formulation) 160 U/kg every other day, prednisolone 5 mg/kg 6 days a week, or placebo, for 19 weeks or until early termination (severe proteinuria or weight gain). The ACTH group demonstrated a significant decrease in spleen weight and total spleen cell counts compared to prednisolone (*p* ≤ 0.001) and placebo (*p* ≤ 0.05). Splenic CD19^+^ B cells were reduced in both the ACTH and prednisolone-treated animals compared to placebo, yet the two treatment arms affected different B cell developmental stages. Only the ACTH-treated mice increased the frequency of immature and T1 B cells. Additionally, while the serum dsDNA autoantibody levels increased steadily during the 19-week treatment period in both the placebo and prednisolone, this was not observed in the ACTH-treated mice [78]. NF-κB signaling is required for B cell differentiation and also regulates the expression of the receptor for B cell activating factor (BAFF or BLyS). ACTH has been previously reported to inhibit NF-κB signaling [78]. Additionally, in a neurological disorder, opsoclonus-myoclonus syndrome (OMS), where BLyS concentration correlated with disease severity, CSF BLyS levels were significantly reduced in patients receiving conventional therapy supplemented with ACTH (−61 %) or corticosteroids (−38 %) [79]. These data taken together suggest a possible role for ACTH in halting the differentiation of autoreactive B cells.

### T Lymphocytes

The most extensive studies evaluating the effects of melanocortins on T cells are done in the contact hypersensitivity skin murine model and the experimental autoimmune uveitis (EAU) murine model [44, 57, 60, 80]. In both studies, α-MSH was able to reduce the levels of IFN-γ from T cells and also increased the regulatory T cell (Treg)-mediated TGF-β1 production. α-MSH-mediated suppression of T cell IFN-γ production was mediated through MC5R [60]. Interestingly, in the EAU model, T cells could be driven to a Treg population by α-MSH [60]. However, in the contact hypersensitivity model, α-MSH was not able to induce Treg without inducing tolerogenic dendritic cells that then drove the Treg population [44].

Tolerogenic dendritic cells (DCs) have been described to induce functional Tregs, and α-MSH-stimulated DCs have been shown to express elevated levels of CD205 and IL-10, characteristic for tolerogenic DCs. Additionally, co-culturing α-MSH-treated DCs with CD4^+^ T cells yielded an expansion of Treg cells, and these α-MSH DC-induced Tregs reduced contact hypersensitivity upon adoptive transfer into sensitized mice [44]. Another study looking at a broader T cell population (CD3^+^) demonstrated that treatment with an α-MSH analogue reduced the ability of DCs to stimulate allogeneic T cells in mixed lymphocyte reactions *in vitro* [47]. Lastly, in an experimental autoimmune uveoretinitis (EAU) mouse model, α-MSH-treated antigen-activated T cells, in an antigen specific manner, suppressed both the incidence and severity of EAU through the conversion of autoantigen-reactive T cells into Tregs [44].

## CARDIOVASCULAR

### Endothelial Cells

Lupus patients are at increased risk of cardiovascular disease [81], and alterations in adhesion molecules, anti-oxidant molecules, and serum lipids are frequently evident. Leukocyte-endothelial cell interaction is a process involving at least three steps: rolling, adhesion, and transmigration into the targeted tissue. These processes are regulated by the expression of cell adhesion molecules (CAMs), including E-selectin, vascular CAM (VCAM)-1, intercellular CAM (ICAM)-1, and leukocyte integrins (Table 2).

**Table 2:** Effects of Melanocortin Peptides on Tissues and Organs

Tissue/Organ		Action	Reference
Skin	Keratinocytes	Increased proliferation Decreased ROS following UVA exposure	[64] [65, 66]
	Fibroblasts	Decreased collagen types I, III, and V Decreased TGF-β Increased superoxide dismutase and hemoxygenase I Reduced ICAM-1	[61, 67]
	Melanocytes	Pigmentation Increased proliferation Decreased apoptosis	[61, 66]
	Dendritic cells	Induce tolerogenic dendritic cells	[44]
Kidney	Podocytes	Reduced foot process effacement	[68]
	Tubular epithelial cells	Reduced apoptosis	[14]
	Kidney	Reduced glomerular damage in AKI model	[14]
CNS	Glia	Reduced apoptosis Decreased cyclooxygenase Decreased iNOS	[39, 53, 69–71]
	Neurons	Indirect survival	[69]
Cardiovascular	Endothelial cells	Downregulation of ICAM-1, VCAM-1, and E-selectin Increased eNOS expression Increased relaxation Increased mitochondrial superoxide dismutase	[48, 50]
	Serum lipids	Decreased serum triglycerides and LDL cholesterol Increased HDL cholesterol	[72–75]

Melanocortins have demonstrated *in vitro* and *in vivo* effects on endothelial cells and CAMs. In human dermal microvascular endothelial cells (HDMECs), α-MSH treatment reduced the mRNA expression of E-selectin, VCAM-1, and ICAM-1 induced by LPS or TNF-α [48]. α-MSH impaired LPS-induced VCAM-1/ICAM-1-mediated lymphocyte adhesion to HDMEC monolayer [48]. Moreover, in a mouse model of LPS-induced cutaneous vasculitis, α-MSH suppressed vascular damage by inhibiting the sustained expression of vascular E-selectin and VCAM-1 [48]. Treatment with α-MSH also improves endothelium-dependent vasodilatation in the mouse aorta by endothelial NO formation. α-MSH increased the expression and phosphorylation of endothelial NO synthase in human cultured endothelial cells [50]. Taken together, these preclinical research findings suggest that melanocortin peptides have the potential to reduce vascular inflammation and damage, potentially modulating deleterious vascular injury in immune-mediated diseases such as SLE.

### Lipids

Studies in patients with kidney disease have demonstrated a potential beneficial effect on serum lipids upon tetracosactide (ACTH1–24) treatment [72, 82]. Of note, 14 patients with idiopathic membranous nephropathy were treated with tetracosactide for 56 days to reduce the level of proteinuria. In addition to therapeutic effect on proteinuria, there was a statistically significant decrease in total serum cholesterol, triglycerides, and LDL cholesterol; an increase in apolipoprotein B; and a corresponding increase in HDL cholesterol and apolipoprotein A1 during the treatment [73].

The tetracosactide effect on serum lipids does not appear to be limited to patients with kidney disease [83]. Thirty male subjects were treated with tetracosactide, cortisol, or placebo for 4 days. Statistically significant decreases in total cholesterol, LDL cholesterol, and apolipoprotein B could be found in the subjects receiving tetracosactide compared to subjects receiving either cortisol or placebo. Additionally, subjects receiving tetracosactide had statistically significant increases in apolipoprotein A1 compared to the other two groups and increased HDL compared to placebo, but not to cortisol. Lowering of triglyceride levels in tetracosactide-treated subjects was observed, but the changes did not reach statistical significance compared to the other groups. These results of melanocortin peptides on serum lipids, while interesting, should be viewed with caution. Tetracosactide or ACTH1–24 is a truncated version of natural ACTH1–39, and there are no definitive studies to suggest a similar effect between the two peptides. Moreover, studies have not been done in patients with SLE or even in animal models of lupus. Therefore it is unclear whether melanocortins could have a beneficial effect on lipid parameters in lupus patients, although the effects with tetracosactide appear relatively consistent between studies.

## SKIN

The pathophysiology of cutaneous manifestations in SLE occurs in several skin cell types and includes immunoglobulin deposition, complement-derived localized damage, and an imbalance between keratinocyte apoptosis and hyperproliferation [6]. α-MSH derives its name from the first studies done demonstrating the ability of α-MSH to increase pigmentation in melanocytes. Subsequent studies have demonstrated that MC1R is the predominant melanocortin receptor identified in a variety of epidermal and dermal cells, including keratinocytes, melanocytes, Langerhans cells, sebocytes, fibroblasts, and endothelial cells [12].

Melanocortins have demonstrated protective effects in various *in vitro* skin models including ultraviolet light exposure and cytokine-mediated inflammation, processes which are implicated in SLE. The skin provides a unique environment where all the components of the POMC system have been identified in various cells of the epidermis [84, 85]. The ability to induce its own melanocortin peptides drives the hypothesis that the melanocortin system has an impact in innate immune responses and epidermal protection from UV exposure, injury, and infection. Melanocortin receptor (MCR) activation also increased melanocyte proliferation and reduced apoptosis [86]. Melanocortin receptor signaling decreased reactive oxygen species production following UVA exposure of keratinocyte cell line *in vitro* [64]. MC1R agonists administered to human fibroblasts *in vitro* decreased collagen type I, III, and V production induced by TGF-β [61, 67] and increased superoxide dismutase and hemoxygenase I, known to protect against oxidative stress, in bleomycin-stimulated fibroblasts *in vitro* [61]. α-MSH significantly attenuated IL-1β-induced IL-8, a neutrophil chemokine, in dermal fibroblasts *in vitro* [87]. Melanocortin receptor agonists and α-MSH have also demonstrated anti-inflammatory and inhibitory effects in a number of inflammatory skin models including atopic dermatitis, psoriasis, and antigen-induced chronic allergic skin inflammation [44, 88, 89].

## KIDNEY

The pathophysiology of glomerular damage associated with SLE is based on immune complex-mediated injury and the cellular inflammatory response to autoantibodies. Damage can occur to mesangial tissue, endothelial tissue in association with capillary wall destruction, or epithelial tissue with direct cytotoxic injury to the podocyte. These patterns of damage combine in various forms and contribute to the histological classification of SLE-associated lupus nephritis [90]. Additionally, direct damage and detachment of podocytes is a significant enough phenomenon in the pathophysiology of lupus nephritis that its assessment is currently being explored as a disease activity marker in lupus nephritis [91].

Clinical research supports the potential utility of melanocortin peptides as treatment for renal symptoms of SLE. The most data in renal disorders exists for the use of melanocortins in idiopathic membranous nephropathy (iMN), a nephrotic syndrome not associated with SLE. iMN is associated with immune complex formation causing subepithelial deposits, membrane thickening, and subsequent inflammatory response [87], similar in pathology to class V lupus nephritis, which is categorized as a membranous glomerulonephritis. Tetracosactide has been used clinically to reduce proteinuria in iMN where steroids were demonstrated to have little clinical benefit [68, 73, 92]. In addition, tetracosactide has been shown to have equivalency to the Ponticelli regimen of a cytotoxic agent and steroids given in alternating months to patients with iMN [93]. There has also been some evidence of clinical benefit in the use of tetracosactide in patients with mesangial glomerulopathies. In one case series, all six patients with mesangioproliferative glomerulopathies and one patient with mesangiocapillary glomerulopathy, all independent of SLE, showed improvement in proteinuria when receiving tetracosactide [92].

The mechanism of improvement in iMN and other renal disorders may be due to immunomodulation but could also be due to a direct protective effect on podocytes [87]. Preclinically, MC1R has been identified and co-localized on podocytes in human kidney tissue [68], and activation of these receptors leads to immunomodulatory and renoprotective effects [94]. The renoprotective effects were evaluated in passive Heymann nephritis model of membranous nephropathy. ACTH, which binds all five melanocortin receptors, including the MC2R that induces steroidogenesis, did not significantly reduce proteinuria [68]. However, proteinuria was significantly reduced in this model by α-MSH, which binds all melanocortin receptors except MC2R, and by the MC1R-specific agonist, MS05. The beneficial effects of α-MSH and MS05 in this model, and the lack of significant effect of ACTH, suggests MC1R agonism, in the absence of MC2R agonism, might protect against kidney damage, on the basis that MC1R agonists decrease measures of oxidative stress [68]. Treatment with an MCR agonist also protected the morphology of the podocyte and significantly decreased effacement and preserved glomerular architecture compared [68].

Additional evidence of melanocortin-driven improvement in renal outcomes was demonstrated in murine lupus models. In one study, 80 % percent of mice treated with placebo required early termination during the 19-week treatment period due to development of severe proteinuria compared to none of the ACTH-treated mice (*p* < 0.05). Histologic assessment demonstrated an overall reduction in renal inflammation and glomerular structural damage, including renal tubular dilation and degeneration, in the ACTH-treated mice [78]. The effects of α-MSH in a separate study demonstrated similar effects on histological preservation and decreased immunoglobulin production [95]. This data suggests that melanocortins may attenuate renal damage associated with lupus.

Melanocortins have also been tested in acute kidney injury models, where pretreatment with α-MSH ameliorated tubular injury score and serum creatinine in the cecal ligation puncture-induced septic kidney injury model [14]. Furthermore, an exploration of the effects of melanocortins on inflamed renal cells *in vitro* revealed that ACTH significantly attenuated apoptosis in renal tubular epithelial cells. The anti-apoptotic effect was attenuated when MC1R was transiently silenced. In addition, renal tubular epithelial cells stimulated with TNF-α exhibit upregulated levels of MC1R protein, suggesting that the protective effects of melanocortin peptides are mediated by at least MC1R [14, 96]. Further research is required to determine the effects of melanocortin peptides in additional animal models of kidney disease and to more firmly establish the receptors and mechanisms of action that might contribute to any protective effects of these peptides against kidney damage and potential preservation of renal function.

## CENTRAL NERVOUS SYSTEM

CNS-associated disease in SLE patients is mainly due to one of two pathophysiologic changes, either thrombotic disease or generalized CNS inflammation. The CNS inflammatory condition is an interesting target when considering melanocortins due to the understanding of their effects in the CNS. Melanocortin receptors are expressed on numerous cells within the CNS, including astrocytes, microglia, endothelial cells, and neurons [39, 69, 70]. Glial cells are the immunocompetent cells of the CNS and, in numerous animal models, strongly influence the pro-inflammatory milieu in injury, infection, and inflammation. Melanocortins attenuated cytokine responses in glial cultures when co-administered with numerous pro-inflammatory agents. In addition, ACTH reduced apoptosis of oligodendroglia in mixed glial cultures stimulated with a variety of cytotoxic agents [39, 69]. Furthermore, α-MSH reduced NO production by decreasing the iNOS expression in rat astrocyte cultures stimulated with LPS and IFN-γ. This inhibitory effect of α-MSH on hypothalamic cellular NO was attributed solely to an effect on iNOS and not the endothelial NO synthase (eNOS) or neuronal NO synthase (nNOS) [71]. Similarly, α-MSH treatment has been shown to downregulate astrocyte cyclooxygenase-2 (COX-2) expression and prostaglandin E2 release [53].

The bioavailability of melanocortin peptides into the CNS might be expected to be limited by their low level of penetrance across the blood-brain barrier (BBB). However, the BBB may be more permeable in SLE, thereby allowing increased bioavailability of circulating melanocortins into the CNS, enabling them to attenuate pro-inflammatory responses and reduce neuronal damage. Furthermore, systemically administered melanocortins can also access melanocortin receptor-rich areas of the intact brain, such as the circumventricular regions, where the BBB is more permeable [97]. In addition, binding melanocortin receptor on peripheral nerve terminals of autonomic nerves may provide a unique neuroimmune modulation [97].

Beyond the direct inflammatory effects of SLE in the CNS, fever is a common constitutional symptom of SLE, fever being a key host response to bacterial invasion aiming at survival of the host [98]. Excessive fever is detrimental to organs, and as such, counter-regulatory processes are in place to modulate the febrile response. This response is thought to occur within the CNS, where febrile and sickness responses are orchestrated [99]. Melanocortin peptides (α-MSH and ACTH) have demonstrated anti-pyretic effects [99–101] and are thought to contribute significantly to these counter-regulatory systems. α-MSH attenuated fever when administered intracerebroventricularly (ICV) 30 min after systemic LPS. In addition, a MC3/4R or a MC4R-selective antagonist (ICV) potentiated the LPS-induced fever and reversed the anti-pyretic effect of ICV α-MSH [98, 99]. Interestingly, though the centrally administered MC3R and MC4R antagonist potentiated LPS fever, it had no effect on systemic ACTH or corticosteroid plasma concentrations [98]. Therefore, the anti-pyretic effects of melanocortins appear to be centrally mediated and independent of adrenal gland stimulation, as similar anti-pyretic effects occur in adrenalectomized rabbits [102]. Melanocortin receptors throughout the brain appear to be neuroprotective both against fever and through their ability to downregulate inflammatory processes occurring in the CNS.

## CONCLUSION

**Fig. 2:**
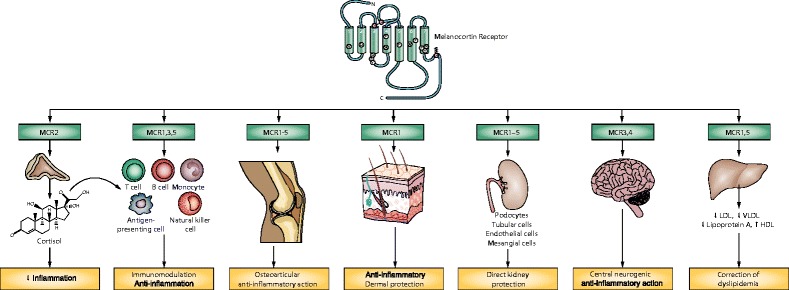
Potential mechanisms of action of melanocortin peptides in the treatment of systemic lupus erythematosus. Melanocortin peptides may reduce disease activity through multiple potential mechanisms, including the following: systemically induced immunosuppression and anti-inflammation in disease-related target organs and tissues secondary to ACTH-induced steroidogenesis, direct effects of MCR activation on systemically induced immunosuppression and anti-inflammation, including regulation of B and T lymphocytes and macrophages, direct effects of MCR activation on hepatic cells to correct dyslipidemia and possibly reduce accelerated atherosclerosis, direct immunosuppressive and anti-inflammatory effects of MCRs expressed in the epidermis and dermis on chronic cutaneous lesions, direct protective effects of MCR activation on kidney cells particularly podocytes, and direct anti-pyretic and neuroprotective effects of MCRs expressed in the central nervous system and central neurogenic anti-inflammation to reduce inflammation-associated neurologic and psychiatric manifestations [94] (reprinted by permission from Macmillan Publishers Ltd).

Melanocortins have the potential to downregulate inflammation in both immune cells and in cells of organs targeted by SLE such as the skin, joints, CNS, and kidney (see Fig. 2) [94]. A number of melanocortin agonists are in early phase development for both their anti-inflammatory properties and their effect on the skin [103]. Interestingly, Acthar Gel, a highly purified preparation of the full-length naturally occurring ACTH (1–39), has been FDA approved for the treatment of SLE since the 1950s and has demonstrated good clinical efficacy in historic literature [33, 34]. The ability to target an endogenous system, which is designed for both modulation of inflammatory processes and organ protection, makes the melanocortin system an ideal receptor target for further development.
